# The HIV self-testing debate: where do we stand?

**DOI:** 10.1186/s12914-018-0146-6

**Published:** 2018-01-18

**Authors:** Marilou Gagnon, Martin French, Yamilee Hébert

**Affiliations:** 1 School of Nursing, Faculty of Health Sciences, University of Ottawa, 451 Smyth Road, Ottawa, ON K1H8M5 Canada; 2 Department of Sociology and Anthropology, Faculty of Arts and Science, Concordia University, 1455 de Maisonneuve Blvd. W. Montréal, Ottawa, H3G 1M8 Canada; 3 Faculty of Medicine, University of Ottawa, 451 Smyth Road, Ottawa, ON K1H8M5 Canada

**Keywords:** AIDS, Arguments, Debate, HIV, Self-testing, Self-test, Home test

## Abstract

**Background:**

Emphasis on HIV testing as a gateway to prevention, treatment and care has grown tremendously over the past decade. In turn, this emphasis on testing has created a demand for new policies, programs, and technologies that can potentially increase access to and uptake of HIV testing. HIV self-testing (HST) technologies have gained important momentum following the approval of the over-the-counter self-tests in the United States, the UK, and France. While the renewed interest in HST has given rise to a number of high quality reviews of empirical studies conducted on this topic, we have yet to find an article that captures the extent of the debate on HST.

**Mapping the debate:**

A critical review of the literature on HST was conducted and organized into three categories based on the focus of the article: 1) Empirical research, 2) Arguments, and 3) Context. We focused exclusively on the second category which included ethical analyses, policy analyses, editorials, opinion pieces, commentaries, letters to the editor and so forth. 10 lines of argument on HST were identified in the literature: 1) Individual – Public Health, 2) Strengths – Limits, 3) Benefits – Harms, 4) Screening – Testing, 5) Target – Market, 6) Health Care – Industry, 7) Regulation – Restriction, 8) Resource-Rich Settings – Resource-Limited Settings, 9) Ethical – Unethical, and 10) Exceptionalism – Normalization. Each line of argument is presented and discussed in the paper.

**Conclusion:**

We conclude by providing examples of critical questions that should be raised in order to take the debate to another level and generate new ways of thinking about HST.

## Background

HIV testing has always been seen as the “keystone” of the HIV response [1]. While this view of HIV testing has not changed significantly over the years, the linkages between testing, prevention, treatment and care have. Emphasis on HIV testing as a gateway to prevention, treatment and care has grown tremendously over the past decade. In turn, this emphasis on testing has created a demand for governments, public health agencies, and HIV organizations to develop new policies, programs, and approaches [2, 3].

This is where HIV self-testing (HST) comes in. This form of testing, which has the potential to increase access to and uptake of HIV testing, has gained important momentum following the approval of the over-the-counter OraQuick® In-Home HIV Test in the United States in 2012 [3, 4], the approval of BioSure® in the UK^1^ in 2015 and the approval of autotest VIH® in France^2^ the same year. However, this approach to HIV testing has also been met with concerns and criticisms, mainly related to accessibility (including access to counseling and health care), accuracy, impact on risk, and potential for misuse or abuse [5]. While a number of high quality reviews of empirical studies have been conducted on HST [3, 6–9], we have yet to find an article that captures the extent of the debate on HST as published in ethical analyses, policy analyses, editorials, opinion pieces, commentaries, letters to the editor, and so forth. This body of literature, which reflects the stance of researchers, policy-makers, health care providers, and people living with HIV, is particularly important for understanding the wider array of factors that determine whether and how HST is integrated into the broader HIV response.

We undertook a critical review of this literature, analyzed its content, and identified key lines of argument that summarize the debate on HST. In this article, we describe these lines of argument and highlight areas in need of further discussion and analysis. We conclude by providing examples of critical questions that should be raised in order to take the debate to another level and generate new ways of thinking about HST.

## Mapping the debate

**Fig. 1:**
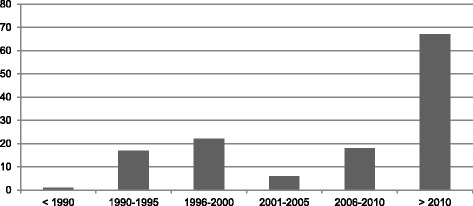
Distribution over the articles sampled over time

**Fig. 2:**
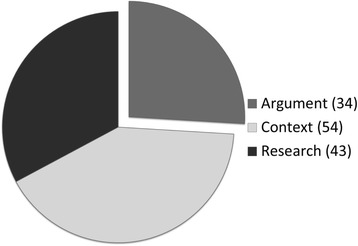
Article distribution

A search of the literature on HST was conducted using the search engines PubMed and CINAHL with key words “self-testing AND HIV”, “self-test AND HIV”, “home testing AND HIV”, and “home test AND HIV” and yielding a final sample of 131 articles (see Table 1 and Fig. 1). We included articles that focused exclusively on HST as “a form of testing in which an individual collects his or her own sample; performs a simple, rapid laboratory test; and is, therefore, the first to know the results” [2, p.126]. As such, we carefully excluded articles that focused on home testing rather than self-testing. While self-testing is often conducted in the home setting, it differs from home testing, i.e., testing conducted in the home setting by a health care provider, or home-sample collection test kits. Yet, these terms are often used interchangeably in the literature. The final sample was organized into three categories based on the focus of the article: 1) Empirical research (*n* = 43; i.e., qualitative studies, quantitative studies, systematic reviews, and literature reviews), 2) Arguments (*n* = 34; i.e., ethical analyses, policy analyses, editorials, opinion pieces, and commentaries), and 3) Context (*n* = 54; i.e., news coverage, reports, and regulatory documents) (see Fig. 2).

**Table 1:** Sample

Key words in title	PubMed	CINAHL	Total
“Self-testing” AND HIV	41	2	43
“Self-test” AND HIV	4	0	4
“Home testing” AND HIV	15	28	43
“Home test” AND HIV	21	20	41
Total	81	50	131

**Fig. 3:**
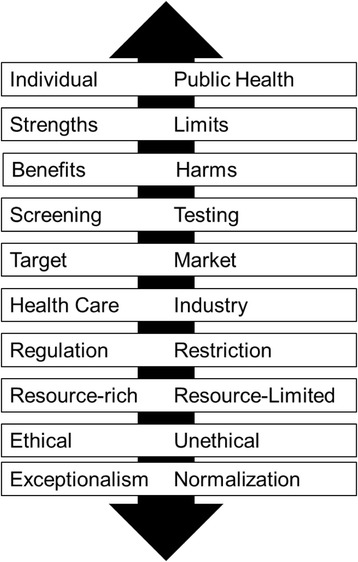
Lines of arguments

This article focuses on the second category, Arguments. This category captures the extent of the debate on HST over the years and includes literature that explored the empirical as well as the ethical, legal, policy, clinical, and public health implications of HST. Surprisingly, literature in this category has been rather marginal in—if not altogether neglected by—other existing reviews of HST. Since our objective was to review, synthesize, and analyze this literature, we opted to conduct a critical review [10]. This type of review “goes beyond mere description of identified articles and includes a degree of analysis and conceptual innovation” [10 p.93]. Each article within this category was read by two members of the research team and then summarized in a table. Over the course of multiple work sessions, the research team discussed each article and assigned codes to every argument presented on HST. These codes were displayed on a whiteboard and organized into broader themes [for example, see [11]. Finally, the themes were examined for potential relationships. We approached the last phase of the analysis with the premise that arguments are dynamic rather static, meaning that they connect together in particular ways and form unique rationales about HST. A total of 10 lines of argument on HST were identified by the research team (see Fig. 3). Each line of argument will be presented and discussed in the following sections.

### Individual – public health

Arguments both for and against HST were clearly located at the intersection between the individual and public health. On one hand, HST was depicted as a large scale public health intervention that has the potential to fill a gap in HIV testing modalities, decrease barriers to HIV testing, increase the number of people who test, increase the number of people who know their status (including those who are infected but unaware of their status), and eventually curb the epidemic primarily through treatment and behavior changes [2, 3, 9, 12–16]. On the other hand, it was clearly located within the realm of the individual – the individual who purchases or obtains the test, does the test, interprets the test, benefits from knowing the results, and acts on the results by seeking care, taking treatment and changing behaviors [2, 3, 9, 12–16]. The importance of “striking a balance” between the individual and public health was a major argument across the literature. It was argued that HST could not just be about the individual or public health [17–20]. Achieving this balance was believed to be essential for the protection of individual rights but most importantly, for the achievements of public health outcomes [17–20]. In other words, it was argued that such outcomes can only be achieved if the individual taking the test and the broader context of HIV treatment and care are given equal attention.

### Strengths – limits

The strengths and limits of HST were both mentioned in the literature, albeit perhaps not equally. The strengths of HST occupied a large portion of the literature and helped define this testing modality as a “game changer”. When compared to provider-administered HIV testing, the strengths of HST included: confidentiality, anonymity, convenience, accessibility, acceptability, satisfaction, safety, and cost-effectiveness [2, 3, 9, 13, 15, 21]. The fact that counseling is not a requirement of HST was seen as an important strength by some authors who considered that counseling could potentially act as a barrier to HIV testing and by others who argued that post-test counseling provided over the phone could be of greater quality than that given in the context of provider-administered testing [12, 18, 22, 23]. In contrast, the lack of counseling requirements was identified as a key limitation of HST along with the fact that HST is not widely accessible and only effective if the “right” individuals engage in testing and are able to access treatment and care following a positive test result – assuming this result is accurate [9, 14, 16, 24–26]. Limitations such as affordability, accuracy, and efficacy were mentioned when authors wanted to question the utility of HST for individuals, and for public health [9, 12, 14, 16, 20, 22, 26]. Emphasis of these limitations also assisted in building a response to the unreserved enthusiasm prompted by the FDA approval of OraQuick® in the United States and the subsequent increase of international considerations for HST.

### Benefits – harms

In the early literature, the potential harms of HST were considered to outweigh the potential benefits. These included the potential psychological harms of getting a positive test result (i.e., fear, anxiety, depression, distress, and suicidal ideation), the potential social harms associated with the wrongful use of the test (i.e., coercion or abuse) and HIV-related stigma more generally, and the potential medical harms caused by a false positive or negative result [5, 9, 17–19, 25, 27, 28]. Over time, more emphasis has been placed on the potential benefits of HST. Two categories of potential benefits have traditionally been put forward by proponents of HST namely individual and public health benefits. HST is believed to benefit individuals, empowering them to take a more active role in managing their health and sexuality as well as making their own choices about when, where, and how to test [2, 3, 18, 23, 24, 26]. Furthermore, HST is believed to produce public health benefits by increasing the number of people who test, the frequency of testing, the number of people who know their status, and the number of people linked to treatment and care – all of which contribute to decreasing HIV transmission [2, 3, 9, 12–16]. Overall, there is a little empirical evidence illustrating either the benefits or the harms of HST [3, 20, 28]. This gap is clearly highlighted in the literature [3]. It is somewhat surprising, therefore, to find that arguments about the benefits of HST have been increasing in frequency in recent years. These arguments commonly present potential benefits as outweighing potential harms, despite the acknowledged lack of empirical evidence to support such a claim.

### Screening – testing

During the analysis, we noted that arguments about HST were typically supported by one of two underlying views concerning the primary function of the test itself. When the potential impact of HST on access to and uptake of HIV testing was discussed in the literature, it was considered to perform the function of a test. In this context, HST was considered to be an accurate and reliable tool for individuals to test themselves where and when they chose [12, 15, 17]. However, some authors warned that HST should not be seen as a substitute for standard laboratory HIV testing [26, 29]. The view that HST could perform as a test but not measure up to the accuracy of a standard laboratory test was commonly expressed by these authors. It was generally agreed that a reactive self-test needs to be followed by a confirmatory laboratory test to reduce false positive results [26]. For this reason, it was argued that HST should be considered a screening rather than a testing tool [3, 12, 15, 17]. This view generally served to emphasize the need for individuals who obtained a positive (or in this case, reactive) result to connect with a health care provider for confirmatory testing, treatment, care and other services. Interestingly, this particular view assumed that individuals who test at home were less likely to follow-up for a confirmation test and medical care if they understood the test as a definitive test rather than a screening tool [15, 24]. The lack of evidence on post-test confirmation and linkage to care in the context of HST [2, 3, 24] is, in large part, responsible for this conceptual tension. This is one area, therefore, that would clearly benefit from further empirical research.

### Target – market

It became evident early in the analysis that specific populations were thought to benefit more than others from HST. These were described as target populations and comprised those who are under-tested, who may not otherwise test, who need to test regularly, who may not have access to provider-administered HIV testing, who are most likely not to know their status, and those at highest risk for HIV [2, 9, 12, 14, 16, 17, 24, 29, 30]. Examples of these populations included sex workers, men who have sex with men, teenagers, young adults, socially disadvantaged groups, minorities, people in rural areas, and people in resource-limited settings. The impact and value of HST was considered to be conditional upon these populations accessing and using the self-test. This could explain why so much emphasis has been placed on acceptability in the empirical literature. It was argued that populations most likely to use HST were not necessarily part of these target populations. In fact, they most likely belonged to what we call “market populations”. Market populations are populations that are segmented not according to risk of exposure to HIV, for example, but rather according to their potential value for the test manufacturer and retailers [24–26, 31]. The literature questioned whether such populations –exemplified, for instance, by the “worried well” and other people at very low risk of HIV – were good or worthy candidates for HST [24–26, 30]. This particular line of argument revealed an interesting interplay between who should test and who is more likely to test. It also revealed that affordability remains an important determinant of access to HST.

### Health care – industry

Our analysis revealed that HST was clearly located at the intersection of health care and industry. Unlike standard HIV testing, which is typically confined within the health care sector, HST is closely tied to industry. As such, concerns were raised that HST was “a middle class model based on the assumption of individuals having sufficient disposable income to buy the test” [18, p.434]. From this perspective, HST was seen primarily as a profitable product for industry, with a secondary potential to contribute to health care. [26]. The importance of lowering the cost of the test was mentioned across the literature with very little acknowledgement of what it would take to achieve this in the for-profit context [3, 12, 14, 16, 19, 24, 26, 32, 33]. The tension between health care and industry was most evident when the issue of public health surveillance was discussed in the literature. The fact that HST was clearly linked to health but located outside the reach of the health care system was considered problematic because it challenged the traditional linkage between testing and public health (surveillance, reporting, and partner notification) [2, 12, 24]. A similar disconnect was noted with post-market surveillance which was seen as contributing to a gap in information on people using the test and the performance of the test itself [2, 12, 24, 30].

### Regulation – restriction

A clear line of argument on the regulation and restriction of HST was identified throughout the literature. Authors believed that state regulation was an essential requirement to achieve quality assurance and protect consumers from defective products [2, 3, 30, 34, 35]. However, they did not support the use of state regulation to restrict access to HST. Setting an age limit for the purchase of the test or selling self-tests behind the counter were highly criticized and seen as restriction rather than regulation [18, 25, 30, 34]. Comparisons between over the counter HIV self-tests and pregnancy tests were often made to illustrate the difference between regulation and restriction. Like the purchase of a pregnancy test, the purchase of an HIV self-test was considered to be a personal decision that must remain free from state intervention [16, 18, 22, 34, 36]. In this sense, it was recommended that the focus of regulation be strictly limited to questions of safety and effectiveness of HIV self-tests [30, 34]. For resource-rich countries like the United States, it was only a matter of determining how to achieve this level of regulation [30]. However, the challenges were viewed as far greater for resource-limited countries both in terms of regulation and restriction [2, 30, 32]. Authors argued that regulatory standards in these countries were likely to differ from resource-rich countries and that existing regulatory systems could pose a problem in the context of HST [30, 32]. They also recommended the use of regulations to protect consumers from low quality tests entering the market and to prevent coercive uses of the test [30, 32].

### Resource-rich settings – resource-limited settings

Arguments about HST took on a particular meaning depending on whether they were being discussed in the context of resource-rich or resource-limited settings. HST in resource-rich settings was seen as less problematic because it was assumed that individuals would be able to pay for the test, do the test, and experience the testing process in a context free from coercion or abuse [30]. It was also the position of many authors that HST was less challenging in resource-rich settings because existing systems ensure proper regulation, access to health care, and protection from abuse [17, 30]. In contrast, HST in resource-limited settings was considered to be more problematic because of numerous issues related to the test itself, namely its cost, accuracy, user-friendliness, and stability in warmer climates, the testing process which requires access to a confirmation test, and the infrastructures needed for regulation, health care and legal protections [15, 17, 30, 32]. In resource-limited settings, the individual doing the test was often seen as more vulnerable, less autonomous, less able to negotiate, and less able to cope with results [17]. That individual was also considered to be less likely to access the necessary treatment and care following a positive test result [17, 32]. Overall, there was a clear divide between arguments about resource-rich and resource-limited settings, with resource-rich settings being seen as more conducive to HST. In our view, more research is necessary to move beyond assumptions about differences between these settings, and to specify the nature of this divide.

### Ethical – unethical

Our analysis revealed two complementary considerations of the ethical implications of HST. Authors considered that HST was ethical when: a) it offered more options, more freedom, more choice, and more power to the individual while contributing to public health benefits, b) it provided the individual with valuable and accurate information about their health and allowed them to do something with that information including accessing health care, notifying partners, taking medications, and changing behaviours, c) it was used safely in a context where the rights and freedoms of the individual are protected, and d) it produced benefits that outweighed its potential harms [2, 9, 17, 18]. However, authors considered that HST was unethical when: a) it increased vulnerabilities and was used to limit the freedom and the rights of the individual, b) access to follow-up confirmation testing, care and treatment could not be guaranteed, c) the necessary infrastructure was not in place to ensure the quality of the test and safeguard human rights, and d) it was seen as “neutral activity” and was taken out of context, thus overlooking the broader social, political, cultural, and legal factors that shape how an individual experiences self-testing and its result [2, 9, 17, 18, 30]. Personal autonomy, which was always mentioned by proponents of HST, was also viewed as fundamentally contextual [17]. As such, it was deemed unethical to implement HST in contexts where individuals lack the autonomy to refuse or accept a self-test [17].

### Exceptionalism – normalization

The literature on HST marked a clear shift away from HIV exceptionalism (i.e., the idea that HIV is different from other diseases and requires different policies and health care approaches) toward normalization (i.e., the idea that HIV is like other diseases and should be addressed as such in policy and health care). Arguments in support of HST were primarily focused on the importance of challenging standards established in the HIV exceptionalism era, especially pre- and post-test counseling standards. From this perspective, the content and format of counseling were seen as contributing to HIV-related stigma by making the test exceptional rather than “normal” [23, 37]. Some considered that counseling practices developed in the exceptionalism era had failed because they were often not done, not done well or not necessary [18, 23]. Some went as far as to say that phone counseling provided by the manufacturers of self-tests was of greater quality than in-person counseling [22, 23]. Resistance to such arguments was found in the earlier literature (1990s) but it gradually disappeared and was replaced with a general acceptance that a shift away from exceptionalism was needed in order to expand HIV testing. In this sense, HST provided equal opportunities to challenge what many described as “outdated” counseling standards, and to remove the stigma associated to the exceptional nature of HIV testing [3, 9, 18, 22, 23, 37]. Comparing the HIV self-test to other self-tests, like the pregnancy test, and making it widely accessible, were considered to be effective strategies to normalize HIV [4, 16, 18, 22]. However, these strategies can also indirectly contribute to further stigma by making HIV testing a “clandestine activity” – one that takes place within the confines of the home and must be hidden from others [24].

## Conclusions

The development and diffusion of HST technology and policy raises a number issues at the intersection of international health policy and human rights, including those related to individual autonomy, access to care, health equality, individual- and population-health, health regulation, and the political economy of biomedical diagnosis. This paper identifies several lines of argument that cross-cut these issues. While our analysis is not exhaustive, it highlights dominant arguments in the current HST debate, providing a useful foundation on which to build further research and analyses as the momentum for this type of testing modality continues to grow. The state of the literature suggests that we have reached a point in our thinking about HST where broader questions need to be raised and new scholarly approaches (including interdisciplinary approaches) need to be explored. These questions and approaches should expand beyond the seemingly neutral terms of using the test, doing the test, offering the test, and scaling-up the test. Raising critical questions about the broader social, cultural, political, legal, economic and human rights issues that HST poses, the strategic positioning of HST in the HIV response, the prevailing assumptions behind the test itself, the ethical implications of HST, the discursive tension between testing and screening, and the challenges of striking a balance between individual and public health as well as between health care and industry is necessary as pressure mounts to make this test widely accessible globally. Throughout the paper, we identified potential avenues for raising such critical questions. It is our hope that these will contribute to the development of new ways of thinking about HST and generate new debates.

## Footnotes

1
https://www.theguardian.com/society/2015/apr/27/hiv-self-testing-kit-on-sale-uk-biosure

2
http://www.huffingtonpost.fr/2015/09/14/auto-test-vih-pharmacie-sida_n_8132574.html
